# Catheter ablation of supraventricular tachycardia in patients with and without structural heart disease: insights from the German ablation registry

**DOI:** 10.1007/s00392-021-01878-z

**Published:** 2021-06-09

**Authors:** Charlotte Eitel, Hüseyin Ince, Johannes Brachmann, Karl-Heinz Kuck, Stephan Willems, Stefan G. Spitzer, Juergen Tebbenjohanns, Leon Iden, Florian Straube, Matthias Hochadel, Jochen Senges, Roland R. Tilz

**Affiliations:** 1 Department of Electrophysiology, University Heart Center Lübeck, Medical Clinic II, University Hospital Schleswig-Holstein, Ratzeburger Allee 160, 23538 Lübeck, Germany; 2 Vivantes Klinika Am Urban und im Friedrichshain und Universitäres Herzzentrum Rostock, Rostock, Germany; 3 Klinikum Coburg, Coburg, Germany; 4 Asklepios Klinik St. Georg, Hamburg, Germany; 5 Universitäres Herzzentrum, Hamburg, Germany; 6 Deutsches Herzzentrum Berlin, Berlin, Germany; 7 Helios Klinikum Hildesheim, Hildesheim, Germany; 8 Segeberger KIiniken, Segeberg, Germany; 9 Munich Clinic Bogenhausen, Munich, Germany; 10 Cardiology, Ludwig-Maximilians-University, Munich, Germany; 11 Stiftung Institut Für Herzinfarktforschung, Ludwigshafen, Germany

**Keywords:** Supraventricular tachycardia, Structural heart disease, Catheter ablation, Registry, Mortality/Survival, Quality and outcomes

## Abstract

**Aim:**

To compare patient characteristics, safety and efficacy of catheter ablation of supraventricular tachycardia (SVT) in patients with and without structural heart disease (SHD) enrolled in the German ablation registry.

**Methods and results:**

From January 2007 until January 2010, a total of 12,536 patients (37.2% with known SHD) were enrolled and followed for at least one year. Patients with SHD more often underwent ablation for atrial flutter (45.8% vs. 20.9%, *p* < 0.001), whereas patients without SHD more often underwent ablation for atrioventricular nodal reentrant tachycardia (30.2% vs. 11.8%, *p* < 0.001) or atrioventricular reentrant tachycardia (9.1% vs. 1.6%, *p* < 0.001). Atrial fibrillation catheter ablation procedures were performed in a similar proportion of patients with and without SHD (38.1% vs. 36.9%, *p* = 0.21).

Overall, periprocedural success rate was high in both groups. Death, myocardial infarction or stroke occurred in 0.2% and 0.1% of patients with and without SHD (*p* = 0.066). Major non-fatal complications prior to discharge were rare and did not differ significantly between patients with and without SHD (0.5% vs. 0.4%, *p* = 0.34). Kaplan–Meier mortality estimate at 1 year demonstrated a significant mortality increase in patients with SHD (2.6% versus 0.7%; *p* < 0.001).

**Conclusion:**

Patients with and without SHD undergoing SVT ablation exhibit similar success rates and low major complication rates, despite disadvantageous baseline characteristics in SHD patients. These data highlight the safety and efficacy of SVT ablation in patients with and without SHD. Nevertheless Kaplan–Meier mortality estimates at 1 year demonstrate a significant mortality increase in patients with SHD, highlighting the importance of treating the underlying condition and reliable anticoagulation if indicated.

**Supplementary Information:**

The online version contains supplementary material available at 10.1007/s00392-021-01878-z.

## Introduction

Supraventricular tachycardia (SVT) comprises a heterogeneous group of arrhythmias with an atrial and/or ventricular rate of more than 100 beats per minute at rest, which involve cardiac tissue at the level of the His bundle or above [1]. The prevalence of SVT is common and high symptom burden often necessitates in-hospital management [2]. Treatment options range from vagal maneuvers, over antiarrhythmic drugs to catheter ablation. Especially during long-term treatment, catheter ablation of SVT has evolved as the treatment of choice. SVT ablation therapy has been shown to be safe and effective and is associated with improved quality of life [2]. Recently published data from the German ablation registry reinforce the value of SVT ablation for long-term symptom improvement in a real-world cohort of 12,566 patients. However, most data on SVT ablation refer to younger patients without SHD. This is also the case in the study by Brachmann et al.[3], in which only a minority of patients undergoing SVT ablation have known SHD. Solely patients undergoing ablation of atrial flutter had known SHD in almost 60% of patients [3]. Furthermore, patients undergoing ablation of atrial flutter had the highest follow-up mortality and stroke rate, partly related to inadequate anticoagulation [3]. One might suggest that patients with SHD have a higher complication and lower success rate potentially due to a more arrhythmogenic substrate. Nevertheless, little is known about differences in success and complication rates of SVT ablation in patients with and without SHD [3, 4].

Therefore, the aim of the following analysis was to assess patient characteristics, outcomes, complications and symptom improvement in a real-world cohort of patients with and without SHD included in a prospective multi-center nationwide registry.

## Methods

### Recruitment and study design

The German ablation registry is a multi-center prospective registry designed to enroll patients undergoing a catheter ablation procedure. A total of 51 German centers collected data of consecutive patients with an age of 18 years or older after written and informed consent was obtained. The registry was approved by the local ethics committees.

From January 2007 until January 2010, a total of 12,536 patients were enrolled in this registry prior to catheter ablation for SVT.

Patients with primary electrical disease, as well as patients undergoing ablation of ventricular arrhythmias were excluded from the present analysis.

### Registry data management and follow-up

The “Institut für Herzinfarktforschung” (IHF, Ludwigshafen, Germany) was responsible for project development and management, as well as data management and clinical monitoring. It also served as the central contract research organization for the study. Participation of the centers was voluntary. The overall concept of the registry and descriptive results of all collected types of SVTs have previously been published [3]. Documentation and data acquisition were voluntary and were carried out on an internet-based case report form system. All site information was confidential, and transmitted data were securely encrypted. The following data were obtained: patient characteristics [age, sex, and co-morbidities, such as hypertension, coronary artery disease, diabetes mellitus, structural heart disease, renal insufficiency, valvular disease, stroke, and the presence of cardiac devices, such as pacemakers (PMs) or implantable cardiac defibrillators (ICDs)], type of SVT ablation, procedural data, and complications during index hospitalization.

After ablation, patients were followed up according to the institutional standard of the treating center. Furthermore, a centralized, prospective one-year follow-up was performed by the IHF based on telephone interviews with special focus on complications, medication, AF symptoms, repeat hospitalizations, arrhythmia recurrences and 12-lead ECG documentation. AF recurrences were defined as documented AF episode lasting at least 30 s. A blanking period was not applied. Clinical symptoms were categorized as unchanged, worsened, or improved.

### Definition of complications

Complications associated with the ablation procedure were categorized into major adverse cardiac events (MACE) including death and myocardial infarction, major non-fatal adverse events, moderate (reversible) and minor adverse events. Major adverse cardiac and cerebrovascular events (MACCE) was defined as a combination of death, myocardial infarction, or stroke. Severe (non-fatal) adverse events included myocardial infarction, stroke, major bleeding, pericardial tamponade, need for emergency cardiac surgery and pulmonary vein stenosis.

### Supraventricular tachycardia ablation procedure

Depending on the underlying diagnosis, modulation or ablation of the slow pathway was performed in patients with atrioventricular nodal reentrant tachycardia (AVNRT), ablation of the accessory pathway in patients with atrioventricular reentrant tachycardia (AVRT) and ablation of the cavotricuspid isthmus with consecutive complete bidirectional block in patients with typical atrial flutter. With respect to catheter ablation of atrial fibrillation (AF), patients underwent circumferential and/or segmental PVI with or without deployment of linear lesions, and/or ablation of complex fractionated atrial electrograms, or ablation of the atrioventricular node.

Procedures and periprocedural management were performed according to the institutional standards of each participating center.

### Statistical analysis

Continuous variables are presented as mean ± standard deviation. For the highly skewed length of hospital stay, median and interquartile range (IQR) are given. Categorical variables are expressed as number and percentage of patients. Differences of categorical distributions were tested for statistical significance using *χ*2 tests, rates of rare events using the Freeman–Halton test. The distributions of continuous variables were compared between two groups (e.g. patients with vs. patients without SHD) using the Mann–Whitney test and between the five patient groups with coronary heart disease, hypertensive heart disease, dilative cardiomyopathy and hypertrophic cardiomyopathy, as well as without heart disease using the Kruskal–Wallis Test. One-year mortality at 366 days after index discharge and cumulative incidence of MACE and MACCE were estimated by the Kaplan–Meier method and compared by the log-rank test. Cox regression was used to calculate hazard ratios with 95% confidence intervals for one-year mortality comparing patients with vs. without SHD, unadjusted and adjusted by including age as a linear term, type of arrhythmia as categorical factor and gender in the model. A *p* value ≤ 0.05 was considered statistically significant. The statistics shown should be regarded as descriptive and were based on the available cases. All analyses were performed at the Biometrics Department of the IHF using the SAS 9.4 software package (SAS Institute, Cary, NC).

## Results

### Patient characteristics

A total of 4660 out of 12,536 patients (37.2%) had known SHD. Patients with SHD were older, more often male and had more co-morbidities as well as previous antiarrhythmic drug failure than patients without SHD. Furthermore, patients with SHD had a higher CHADS_2_-Score (2.8 ± 1.5 vs. 1.4 ± 1.2, *p* < 0.001) and more often received oral anticoagulation (41.1% vs. 22.2%, *p* < 0.001). 53.3% of patients with SHD had coronary heart disease, 31.8% hypertensive heart disease, 20.8% valvular heart disease, 8.8% dilative cardiomyopathy and 1.7% hypertrophic cardiomyopathy. Detailed patient characteristics are shown in Table 1. Furthermore, characteristics were separately analyzed for AF and non-AF ablation procedures (supplemental Table 1 and 4).

**Table 1:** Characteristics of patients with and without structural heart disease

	Patients with SHD *n* = 4664	Patients without SHD *n* = 7872	*p* value
Age* (years), mean ± SD	66.3 ± 10.4	55.8 ± 14.8	** < 0.001**
Age > 75 years, %	17.1	5.0	** < 0.001**
Male, %	74.3	54.4	** < 0.001**
Antiarrhythmic drug failure, %	72.0	54.3	** < 0.001**
Cardiac disease			
Coronary artery disease, %	53.3	0	** < 0.001**
Prior myocardial infarction, %	14	0	** < 0.001**
Cardiomyopathy, %	10.4	0	** < 0.001**
Hypertrophic cardiomyopathy, %	15.8	0	
Dilative cardiomyopathy, %	84.2	0	
Hypertensive heart disease, %	31.8	0	** < 0.001**
Valvular heart disease, %	20.8	0	** < 0.001**
Comorbidities			
Diabetes mellitus, %	17.0	6.3	** < 0.001**
Arterial hypertension*, %	73.3	41.6	** < 0.001**
Renal failure*, %	10.7	1.7	** < 0.00**1
Previous stroke*, %	4.4	2.8	0.069
Devices (PM, ICD,CRT), %	15.1	3.1	** < 0.001**
Left ventricular ejection fraction			** < 0.001**
Normal (> 50%), %	63.7	94.6	
Mildly reduced (41–50%), %	19.7	4.2	
Reduced (< 40%), %	16.6	1.2	
CHADS_2_-Score*, mean ± SD	2.8 ± 1.5	1.4 ± 1.2	** < 0.001**
Oral anticoagulation, %	41.1	22.2	** < 0.001**

A *p* value ≤ 0.05 was considered statistically significant

*Data available in 14% of patients due to later inclusion of the variable in the study

*CRT* cardiac resynchronization therapy, *ICD* implanted cardioverter defibrillator, *PM* pacemaker, *SD* standard deviation, *SHD* structural heart disease

### Procedural data and periprocedural complications

**Fig. 1:**
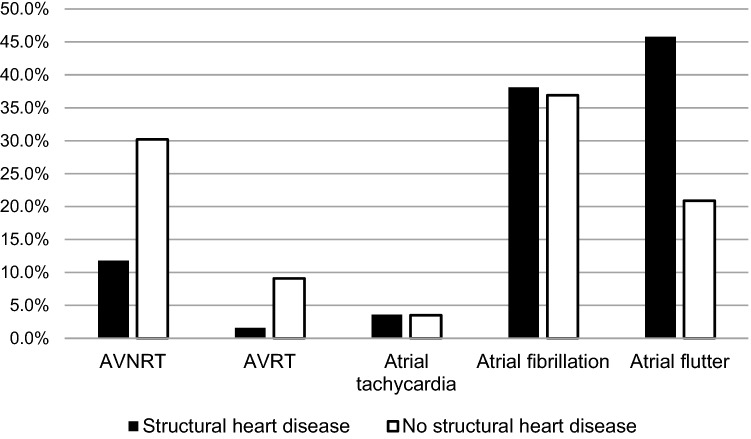
Type of supraventricular tachycardia ablated in patients with and without structural heart disease. *p* < 0.001 for AVNRT, AVRT and atrial flutter. AVNRT: atrioventricular nodal reentrant tachycardia, AVRT: atrioventricular reentrant tachycardia

**Fig. 2:**
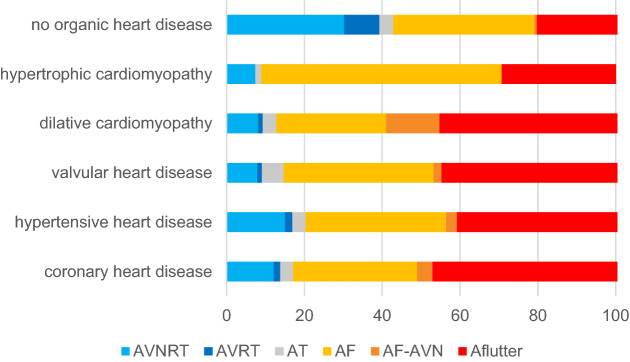
Percentage of supraventricular tachycardia ablation performed according to underlying heart disease. AF: atrial fibrillation, AF-AVN: atrial fibrillation—atrioventricular node ablation, Aflutter: atrial flutter, AT: atrial tachycardia, AVNRT: atrioventricular nodal reentrant tachycardia, AVRT: atrioventricular reentrant tachycardia

With respect to ablation procedure performed, patients with SHD more often underwent ablation for atrial flutter (45.8% vs. 20.9%, *p* < 0.001), whereas patients without SHD more often underwent catheter ablation for AVNRT (30.2% vs. 11.8%, *p* < 0.001) or AVRT (9.1% vs. 1.6%, *p* < 0.001). Atrial tachycardia (3.6% vs. 3.5%, *p* = 0.65) and AF (38.1% vs. 36.9%, *p* = 0.21) catheter ablation procedures were performed in a similar proportion of patients with and without SHD, while ablation of the atrioventricular node was performed more frequently in patients with SHD (4.0% vs. 0.6%, *p* < 0.001) (Fig. 1). Further differentiation of type of SVT ablation performed according to underlying heart disease is illustrated in Fig. 2.

Procedure duration was shorter in patients with SHD, while fluoroscopy time, dose area product and cumulative duration of all applications were longer in patients with SHD (Table 2).

**Table 2:** Procedural data and periprocedural complications in patients with and without structural heart disease

	Patients with SHD *n* = 4664	Patients without SHD *n* = 7872	*p* value
De novo ablation, %	88.4	88.7	0.64
Procedure duration (min), median (IQR)	100 (60; 165)	110 (62; 170)	** < 0.001**
Fluoroscopy time (min), median (IQR)	18 (10; 32)	17 (8; 30)	** < 0.001**
Dose area product [(cGy)*cm^2^], median (IQR)	2428 (1026; 5323)	1771 (664; 4280)	** < 0.001**
Cumulative duration of all applications (seconds), median (IQR)	607 (300; 1631)	457 (166; 1615)	** < 0.001**
Death, *n* (%)	4 (0.1)	0 (0.0)	**0.019**
MACE (death, myocardial infarction), *n* (%)	4 (0.1)	3 (0.0)	0.44
MACCE (death, myocardial infarction, stroke), *n* (%)	10 (0.2)	6 (0.1)	**0.066**
Nonfatal Stroke, *n* (%)	6 (0.1)	4 (0.1)	0.19
Major bleeding (intervention), *n* (%)	19 (0.4)	27 (0.3)	0.55
Transient ischemic attack, *n* (%)	3 (0.1)	3 (0.0)	0.68
Cardiac tamponade, *n* (%)	21 (0.5)	35 (0.5)	1.0
Aneurysm spurium, arteriovenous fistula, *n* (%)	41 (0.7)	47 (0.6)	0.08
Atrio-esophageal fistula, *n* (%)	0	0	0
Minor bleeding (without intervention), *n* (%)	79 (1.7)	108 (1.4)	0.17
Duration of in-hospital stay, days	3 (2;6)	2 (2;4)	** < 0.001**
Arrhythmia recurrence (in-hospital), *n* (%)	173 (3.7)	279 (3.5)	0.63

A *p* value ≤ 0.05 was considered statistically significant

*IQR* interquartile range

Overall acute success rates were high (95.8% vs. 96.6%, *p* = 0.027) in patients with and without SHD.

Death, myocardial infarction or stroke (MACCE) occurred in 10 patients (0.2%) with and 6 patients (0.1%) without SHD (*p* = 0.066). Other major complications prior to discharge were rare (57/12523, *p* = 0.4%) without difference between patient groups (0.5% vs. 0.4%, *p* = 0.34) (Table 2).

Procedural data were separately analyzed for AF and non-AF ablation procedures (supplemental Table 2 and 5).

### Follow-Up

#### Arrhythmia recurrence and symptoms

**Fig. 3:**
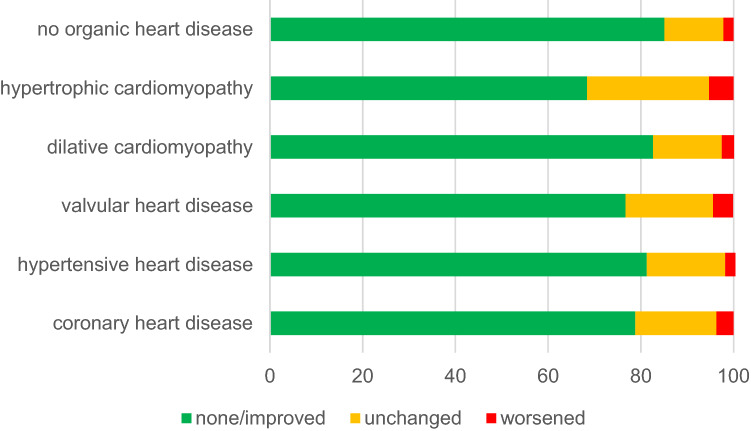
Change in symptoms following supraventricular tachycardia ablation according to underlying heart disease

Recurrence rate during a follow-up period of 551.7 ± 138.8 and 572.8 ± 163.4 days (*p* < 0.001) did not differ in patients with or without SHD (33.5% vs. 32.1%, *p* = 0.12) with reablations being performed in a similar proportion of patients (14.6% vs. 14.0%, *p* = 0.37) (Table 3). However, patients with SHD less often showed improvement of symptoms or no symptoms (79.2% vs. 85.1%, *p* < 0.001). Further analysis of symptom course according to underlying heart disease reveals that patients with hypertrophic cardiomyopathy, valvular heart disease and coronary heart disease experience least improvement of symptoms with 5.3% of hypertrophic cardiomyopathy patients, 4.3% of valvular heart disease patients and 3.7% of coronary heart disease patients even experiencing worsening of symptoms compared to 2.7% with hypertensive heart disease and 2.6% with dilative cardiomyopathy, as well as 2.2% of patients without SHD, respectively (Fig. 3).

**Table 3:** Twelve-month follow-up of patients with and without structural heart disease

	Patients with SHD *n* = 4664	Patients without SHD *n* = 7872	*p* value
Follow-up completed, *n* (%)	4555 (0.98)	7561 (0.96)	** < 0.001**
Documented arrhythmia recurrence, *n* (%)	1335 (31.4)	2166 (29.6)	**0.044**
Rehospitalization, *n* (%)	1911 (47.4)	2523 (36.1)	** < 0.001**
Re-ablation, *n* (%)	622 (14.6)	1023 (14.0)	0.34

A *p* value ≤ 0.05 was considered statistically significant

#### Adverse events and mortality

**Fig. 4:**
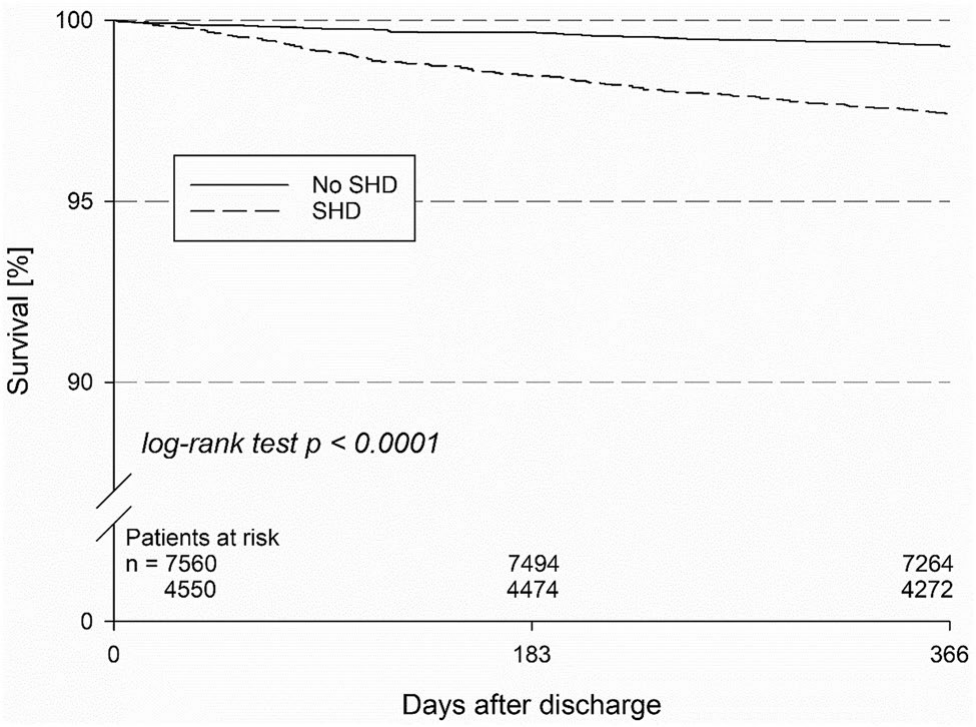
Kaplan–Meier mortality estimate at 1 year demonstrated a significant mortality increase in patients with SHD (2.6% vs. 0.7%; *p* < 0.001)

Kaplan–Meier mortality estimate at 1 year demonstrated a significant mortality increase in patients with SHD (2.6% vs. 0.7%; *p* < 0.001; Fig. 4). Cox regression analysis was performed with a calculated hazard ratio of 3.62 (95%-CI 2.63–4.98) for 1-year-mortality. After adjustment for age, gender and type of arrhythmia SHD independently increased the risk of all-cause mortality with an adjusted hazard ratio of 2.04 (1.45–2.87; *p* < 0.001). Furthermore, rate of MACE (2.9% vs. 0.8%, *p* < 0.001) and MACCE (3.6% vs. 1.2%, *p* < 0.001) was significantly higher in patients with SHD. With respect to non-fatal adverse events, patients with SHD more often experienced myocardial infarction (0.6% vs. 0.2%, *p* < 0.001), stroke (1.0% vs. 0.6%, *p* = 0.016), transient ischemic attack (0.7% vs. 0.3%, *p* = 0.002) and major bleeding (1.0% vs. 0.6%, *p* = 0.019). Further analysis according to AF and non-AF ablation procedures is shown in supplemental Tables 3 and 6.

## Discussion

### Main findings of the study

The main findings of our analysis of 12,536 patients with (37.2%) and without SHD (62.8%) undergoing SVT ablation within the multi-center German ablation registry can be summarized as follows: (1) Patients with SHD differ with respect to baseline characteristics (older, more co-morbidities, higher amount of previous antiarrhythmic drug failure) and ablation procedure performed (more patients undergoing ablation for atrial flutter and ablation of the atrioventricular node for AF treatment). (2) Overall acute success rates were slightly lower in patients with versus without SHD, while complication rates were similar. (3.) During one-year follow-up, mortality and rate of MACCE were higher in patients with SHD.

### Periprocedural outcome

The high success rate of SVT ablation in the German ablation registry, that is equal to or slightly higher than in other registries [5–7] has already been discussed by Brachmann et al. and been attributed to advancements in catheter ablation technologies [3]. Our study further extends these findings to patients with and without SHD who slightly differ with respect to acute success rates (95.8% vs. 96.6%, 0.037), probably due to disadvantageous baseline characteristics. Furthermore, major non-fatal complication rates were similarly low in patients with and without SHD (0.5% vs. 0.4%, 0.34). The slightly higher rate of MACCE in patients with SHD (0.2% vs. 0.1%; *p* = 0.066) was mainly driven by a higher death rate with three cardiac, one non-cardiac and one sudden death in SHD patients vs. none in patients without SHD (*p* < 0.019). This may be explained by a higher age at baseline with 17.1% of SHD patients being older than 75 years vs. 5.0% of patients without SHD (*p* < 0.001), a worse left ventricular ejection fraction (LVEF) with 16.6% having an LVEF of less than 40% vs. 1.2% in patients without SHD (*p* < 0.001) and a higher number of co-morbidities.

### Analysis of SHD patients according to underlying heart disease

As noted above, patients with SHD more often underwent ablation for atrial flutter (45.8% vs. 20.9%, *p* < 0.001), and the atrioventricular node for AF treatment (4.0% vs. 0.6%, *p* < 0.001).

A more detailed analysis according to underlying heart disease reveals that a high proportion of patients with hypertrophic cardiomyopathy (61.8%) underwent AF catheter ablation. This is related to the fact that AF is the most frequent arrhythmia in hypertrophic cardiomyopathy patients and that rhythm control in these patients may be particularly beneficial for symptom control and hemodynamic improvement [8, 9]. However, arrhythmia recurrences are frequent going along with less improvement of symptoms during follow-up [10]. Arrhythmia recurrence rate of hypertrophic cardiomyopathy patients was 42.6% in this registry and went along with a low rate of symptom improvement in only 50.9% of patients.

Patients with dilative cardiomyopathy were most likely to undergo AV node ablation for AF treatment. Previous analysis of data of the German ablation registry with respect to AF ablation and AV nodal ablation showed that during the inclusion period of this registry (2007–2010) almost 50% of heart failure patients with reduced LVEF underwent AV nodal ablation [11]. This was most likely related to a higher number of co-morbidities, older age, more impaired LVEF and higher NYHA class [11, 12]. Furthermore, these patients more often presented with permanent or long-standing persistent AF and implanted devices [11, 12]. Nevertheless, recent data, like the CASTLE-AF trial highlight the value of pulmonary vein isolation in patients with HF [13]. In this study, 363 patients with AF and HF were randomized to PVI or conventional treatment (rate or medical rhythm control). Over a follow-up of 60 months there was a significant reduction of the primary endpoint of all-cause mortality [HR 0.53 (95% CI, 0.32–0.86), *p* = 0.011; log-rank test: *p* = 0.009] and worsening HF admissions [HR 0.56 (95% CI, 0.37–0.83), *p* = 0.004; Log-rank test: *p* = 0.004] in patients undergoing PVI. The AMICA trial further supports the hypothesis that ablation may be more beneficial in patients with less advanced HF as included in AMICA [14].

### Long-term follow-up

During a follow-up period of 551.7 ± 138.8 and 572.8 ± 163.4 days (*p* < 0.001), patients with and without SHD did not differ with respect to arrhythmia recurrences (33.5% vs. 32.1%, *p* = 0.12) or reablations (14.6% vs. 14.0%, *p* = 0.37). However, patients with SHD less often showed improvement of symptoms or no symptoms (79.2% vs. 85.1%, *p* < 0.001). The subgroup of patients with hypertrophic cardiomyopathy, valvular heart disease and coronary heart disease experienced least improvement of symptoms or even worsening of symptoms. This may partly relate to the underlying heart disease and partly to a higher recurrence rate in patients with valvular heart disease (42.2%) and hypertrophic cardiomyopathy (42.6%).

Kaplan–Meier mortality estimate at 1 year demonstrated a significant mortality increase in patients with SHD (2.6% vs. 0.7%; *p* < 0.001). Furthermore, rates of MACE (2.9% vs. 0.8%, *p* < 0.001) and MACCE (3.6% vs. 1.2%, *p* < 0.001) as well as non-fatal adverse events were significantly higher in patients with SHD. This may probably be related to the above-mentioned worse baseline characteristics, as well as the higher prevalence of atrial flutter going along with silent atrial fibrillation and a higher risk of stroke and mortality.

### Limitations

Limitations of this analysis relate to the non-randomized study design with prospectively assessed registry data. Nevertheless, analyses of registries are of importance to assess ablation strategies and outcome in the general population managed in clinical practice. Voluntary participation might potentially go along with underreporting of procedural complications or recurrences. Recurrences might also have been missed due to lack of centralized systematic rhythm follow-up with Holter ECGs. Instead, follow-up care was left at the discretion of the treating center and follow-up data were assessed by telephone interview 12 months after the ablation procedure and a 12-lead ECG with all patients being independently contacted by the IHF.

## Conclusion

Patients with and without SHD undergoing SVT ablation within the German ablation registry exhibit high overall success rates and low complication rates, despite higher age and more co-morbidities in SHD patients. These data highlight the safety and efficacy of SVT ablation in patients with and without SHD. Nevertheless, Kaplan–Meier mortality estimates at 1 year demonstrate a significant mortality increase in patients with SHD, highlighting the importance of treating the underlying condition and reliable anticoagulation if indicated.

## Supplementary Information

Below is the link to the electronic supplementary material.

Supplementary file1 (DOCX 227 kb)

Supplementary file2 (DOCX 27 kb)
