# Supplementary material for: Catheter ablation of supraventricular tachycardia in patients with and without structural heart disease: insights from the German ablation registry
Source: Clin Res Cardiol. 2021 Jun 9;111(5):522–9. doi: 10.1007/s00392-021-01878-z (PMC9054935; doi:10.1007/s00392-021-01878-z)
Supplement: Supplementary file 2 — Supplementary file2 (DOCX 27 kb) [file 392_2021_1878_MOESM2_ESM.docx]

**Supplemental Tables**

**Supplemental Table 1:** Characteristics of SVT patients (AF patients excluded) with and without structural heart disease.

|  | **Patients with SHD**  **n=2889** | **Patients without SHD**  **n=4965** | **p-value** |
| --- | --- | --- | --- |
| Age* (years), mean +/-SD | 67.4±10.8 | 53.7±16.4 | **<0.001** |
| Age>75 years, % | 22.2 | 6.1 | **<0.001** |
| Male, % | 73.1 | 47.7 | **<0.001** |
| Antiarrhythmic drug failure, % | 65.9 | 54.4 | **<0.001** |
| **Cardiac disease** |  |  |  |
| Coronary artery disease, % | 55.3 | 0 | **<0.001** |
| Prior myocardial infarction, % | 13.9 | 0 | **<0.001** |
| Cardiomyopathy, % | 9.5 | 0 | **<0.001** |
| Hypertrophic cardiomyopathy, % | 11.3 | 0 |  |
| Dilative cardiomyopathy, % | 88.7 | 0 |  |
| Hypertensive heart disease, % | 31.5 | 0 | **<0.001** |
| Valvular heart disease, % | 20.2 | 0 | **<0.001** |
| **Comorbidities** |  |  |  |
| Diabetes mellitus, % | 19.2 | 6.8 | **<0.001** |
| Arterial hypertension*, % | 72.5 | 33.9 | **<0.001** |
| Renal failure*, % | 13.1 | 1.6 | **<0.00**1 |
| Previous stroke*, % | 4.7 | 0.9 | <0.001 |
| Devices (PM, ICD,CRT), % | 13.6 | 2.7 | **<0.001** |
| Left ventricular ejection fraction |  |  | **<0.001** |
| Normal (>50%), % | 61.4 | 95.4 |  |
| Mildly reduced (41-50%), % | 20.6 | 3.5 |  |
| Reduced (<40%), % | 18.1 | 1.2 |  |
| CHADS_2_-Score*, mean +/- SD | 1.4±1.0 | 0.5±0.7 | **<0.001** |
| Oral anticoagulation, % | 54.6 | 19.2 | **<0.001** |

- *Data available in 14% of patients due to later inclusion of the variable in the study.
- CRT: cardiac resynchronization therapy; ICD: implanted cardioverter defibrillator; PM: pacemaker; SD: standard deviation; SHD: structural heart disease.

**Supplemenal Table 2:** Procedural data and periprocedural complications in SVT patients (AF patients excluded) with and without structural heart disease.

|  | **Patients with SHD**  **n=2887** | **Patients without SHD**  **n=4965** | **p-value** |
| --- | --- | --- | --- |
| De novo ablation, % | 91.5 | 92.7 | **0.043** |
| Procedure duration (min), median (IQR) | 75 (50; 115) | 80 (55; 120) | **0.001** |
| Fluoroscopy time (min), median (IQR) | 14 (8; 24) | 11 (6; 20) | **0.001** |
| Dose area product [(cGy)*cm^2^], median (IQR) | 1853 (796; 4233) | 1075 (400; 2550) | **<0.001** |
| Cumulative duration of all applications (seconds), median (IQR) | 445 (250; 860) | 258 (120; 584) | **<0.001** |
| Death, n (%) | 2 (0.1) | 0 (0.0) | 0.14 |
| MACE (death, myocardial infarction), n (%) | 2 (0.1) | 2 (0.0) | 0.64 |
| MACCE (death, myocardial infarction, stroke), n (%) | 2 (0.1) | 1 (0.0) | 0.56 |
| Nonfatal Stroke, n (%) | 0 (0.0) | 1 (0.0) | 1.0 |
| Major bleeding (intervention), n (%) | 3 (0.1) | 6 (0.1) | 1.0 |
| Transient ischemic attack, n (%) | 0 (0.0) | 1 (0.0) | 1.0 |
| Cardiac Tamponade, n (%) | 3 (0.1) | 13 (0.3) | 0.19 |
| Aneurysm spurium, arteriovenous fistula, n (%) | 19 (0.7) | 17 (0.3) | 0.056 |
| Atrio-esophageal fistula, n (%) | 0 | 0 | 0 |
| Minor bleeding (without intervention), n (%) | 31 (1.1) | 20 (0.4) | **<0.001** |
| Duration of in-hospital stay, days | 3 (2;6) | 2 (2;3) | **<0.001** |
| Arrhythmia recurrence (in-hospital), n (%) | 50 (1.7) | 66 (1.3) | 0.16 |

- IQR: interquartile range.

**Supplemental Table 3:** Twelve-month follow-up of SVT patients (AF patients excluded) with and without structural heart disease.

|  | **Patients with SHD**  **n=2889** | **Patients without SHD**  **n=4965** | **p-value** |
| --- | --- | --- | --- |
| Follow-up completed, n (%) | 2820 (0.98) | 4721 (0.95) | **<0.001** |
| Documented arrhythmia recurrence, n (%) | 629 (24.3) | 959 (21.2) | **0.002** |
| Rehospitalization, n (%) | 1114 (45.8) | 1355 (31.4) | **<0.001** |
| Re-ablation, n (%) | 262 (10.1) | 408 (9.0) | 0.11 |

**Supplemental Table 4:** Characteristics of AF patients only with and without structural heart disease.

|  | **Patients with SHD**  **n=1775** | **Patients without SHD**  **n=2907** | **p-value** |
| --- | --- | --- | --- |
| Age* (years), mean +/-SD | 64.4±9.3 | 59.2±10.8 | **<0.001** |
| Age>75 years, % | 8.8 | 3.1 | **<0.001** |
| Male, % | 70.3 | 65.7 | **<0.001** |
| Antiarrhythmic drug failure, % | 90.0 | 88.2 | 0.067 |
| **Cardiac disease** |  |  |  |
| Coronary artery disease, % | 50.2 | 0 | **<0.001** |
| Prior myocardial infarction, % | 14.1 | 0 | **<0.001** |
| Cardiomyopathy, % | 11.9 | 0 | **<0.001** |
| Hypertrophic cardiomyopathy, % | 21.7 | 0 |  |
| Dilative cardiomyopathy, % | 78.3 | 0 |  |
| Hypertensive heart disease, % | 32.3 | 0 | **<0.001** |
| Valvular heart disease, % | 21.8 | 0 | **<0.001** |
| **Comorbidities** |  |  |  |
| Diabetes mellitus, % | 13.4 | 5.3 | **<0.001** |
| Arterial hypertension*, % | 74.3 | 55.1 | **<0.001** |
| Renal failure*, % | 7.6 | 1.7 | **<0.00**1 |
| Previous stroke*, % | 4.0 | 6.3 | 0.17 |
| Devices (PM, ICD,CRT), % | 17.7 | 5.3 | **<0.001** |
| Left ventricular ejection fraction |  |  | **<0.001** |
| Normal (>50%), % | 67.7 | 93.3 |  |
| Mildly reduced (41-50%), % | 18.2 | 5.4 |  |
| Reduced (<40%), % | 14.1 | 1.3 |  |
| CHADS_2_-Score*, mean +/- SD | 1.2±0.9 | 0.8±0.8 | **<0.001** |
| Oral anticoagulation, % | 90.2 | 89.4 | 0.42 |

- *Data available in 14% of patients due to later inclusion of the variable in the study.
- CRT: cardiac resynchronization therapy; ICD: implanted cardioverter defibrillator; PM: pacemaker; SD: standard deviation; SHD: structural heart disease.

**Supplemental Table 5:** Procedural data and periprocedural complications in of AF patients only with and without structural heart disease.

|  | **Patients with SHD**  **n=1775** | **Patients without SHD**  **N=2907** | **p-value** |
| --- | --- | --- | --- |
| De novo ablation, % | 83.4 | 81.7 | 0.15 |
| Procedure duration (min), median (IQR) | 165 (120; 210) | 173 (125; 215) | **0.003** |
| Fluoroscopy time (min), median (IQR) | 27 (17; 46) | 28 (18; 43) | 0.27 |
| Dose area product [(cGy)*cm^2^], median (IQR) | 3747 (1788; 7182) | 3410 (1757; 6667) | 0.099 |
| Cumulative duration of all applications (seconds), median (IQR) | 2340 (966; 3420) | 2400 (1392; 3600) | **<0.001** |
| Death, n (%) | 2 (0.1) | 0 (0.0) | 0.14 |
| MACE (death, myocardial infarction), n (%) | 2 (0.1) | 2 (0.1) | 0.64 |
| MACCE (death, myocardial infarction, stroke), n (%) | 8 (0.5) | 5 (0.2) | 0.091 |
| Nonfatal Stroke, n (%) | 6 (0.3) | 3 (0.1) | 0.091 |
| Major bleeding (intervention), n (%) | 16 (0.9) | 21 (0.7) | 0.50 |
| Transient ischemic attack, n (%) | 3 (0.2) | 2 (0.1) | 0.37 |
| Cardiac Tamponade, n (%) | 18 (1.2) | 22 (0.9) | 0.41 |
| Aneurysm spurium, arteriovenous fistula, n (%) | 22 (1.2) | 30 (1.0) | 0.57 |
| Atrio-esophageal fistula, n (%) | 0 | 0 | 0 |
| Minor bleeding (without intervention), n (%) | 48 (2.7) | 88 (3.0) | 0.59 |
| Duration of in-hospital stay, days | 4 (2;6) | 3 (2;5) | **<0.001** |
| Arrhythmia recurrence (in-hospital), n (%) | 123 (6.9) | 213 (7.3) | 0.61 |

- IQR: interquartile range.

**Supplemental Table 6:** Twelve-month follow-up of of AF patients only with and without structural heart disease.

|  | **Patients with SHD**  **n=1775** | **Patients without SHD**  **n=2907** | **p-value** |
| --- | --- | --- | --- |
| Follow-up completed, n (%) | 1735 (0.98) | 2840 (0.98) | 0.72 |
| Documented arrhythmia recurrence, n (%) | 706 (42.3) | 1207 (43.3) | 0.49 |
| Rehospitalization, n (%) | 797 (49.7) | 1168 (43.64) | **<0.001** |
| Re-ablation, n (%) | 360 (21.5) | 615 (22.1) | 0.67 |
